# Distinct clinical and inflammatory signatures in anterior vs. posterior circulation strokes

**DOI:** 10.3389/fmed.2025.1613282

**Published:** 2026-01-21

**Authors:** Chen Hanna Ryder, Carmit Gal, Gili Barkay, Michael Zagorodniuk, Dan Paz, Galina Keigler, Einav Levy, Yori Gidron, Mohammad Ebrahim Naffaa, Samih Badarny

**Affiliations:** 1 Western Galilee College, Brain and Behavior Research Institute, Acre, Israel; 2 Department of Psychology, Kinneret College on the Sea of Galilee, Jordan Valley, Israel; 3 Department of Neurology, Galilee Medical Center, Nahariyya, Israel; 4 Azrieli Faculty of Medicine, Bar-Ilan University, Safed, Israel; 5 MRI Unit, Department of Radiology, Galilee Medical Center, Nahariyya, Israel; 6 Research Center for Innovation in Social Work, Tel Hai College, Qiryat Shemona, Israel; 7 Faculty of Welfare and Health Sciences, University of Haifa, Haifa, Israel; 8 Rheumatology Unit, Galilee Medical Center, Nahariyya, Israel

**Keywords:** anterior circulation (AC), posterior circulation (PC), stroke severity, ischemic stroke, stroke severity (NIHSS), C-reactive protein (CRP), white blood cells (WBC), precision medicine

## Abstract

**Background:**

While clinical differences between anterior (AC) and posterior (PC) circulation strokes are recognized, the underlying biological distinctions remain poorly defined, limiting the development of personalized therapies. This study aimed to delineate the unique clinical profiles and identify the distinct inflammatory signatures of AC versus PC ischemic strokes, thereby providing a biological basis for their classification as separate pathophysiological entities.

**Methods:**

This retrospective cohort study analyzed 499 ischemic stroke patients (434 AC, 65 PC) admitted to a tertiary neurological center. Stroke subtype was confirmed by neuroimaging. A comprehensive comparative analysis was conducted on demographic characteristics, vascular risk factors, clinical severity via National Institutes of Health Stroke Scale (NIHSS) scores, and key laboratory parameters upon admission, with a focus on inflammatory markers (C-reactive protein and white blood cell count).

**Results:**

The core innovation of this study is the discovery of a novel, divergent inflammatory signature: AC strokes were characterized by a systemic, C-reactive protein (CRP)-dominant response (14.97 vs. 8.65 mg/L, *p* < 0.001), whereas PC strokes exhibited a distinct, leukocyte-dominant profile (WBC count 10.80 vs. 9.36 × 10^9^/L, *p* < 0.001). This biological divergence was mirrored by a notable clinical dissociation, where PC strokes presented with significantly lower baseline NIHSS scores (8.31 vs. 13.47, *p* < 0.001), highlighting the insensitivity of standard severity scales to posterior-specific deficits. Furthermore, PC strokes were associated with older age and a higher rate of previous stroke, while AC strokes were more frequently linked to atrial fibrillation.

**Conclusion:**

Our findings establish that AC and PC strokes are not merely anatomical variants but distinct pathophysiological entities, each possessing a unique biological fingerprint. The discovery of opposing inflammatory profiles, coupled with the clear dissociation from standard clinical severity scores, provides a compelling rationale to move beyond a “one-size-fits-all” approach in stroke care. This evidence lays a critical foundation for developing subtype-specific diagnostic biomarkers and targeted immunomodulatory strategies, representing a key translational step toward advancing precision medicine for stroke patients.

## 1 Introduction

Acute ischemic stroke (AIS) remains one of the leading causes of mortality and long-term disability worldwide, imposing a significant burden on individuals, families, and healthcare systems (1). Stroke is not a single disease entity but a heterogeneous clinical syndrome with diverse etiologies, risk factors, and manifestations (2) While nearly 87% of all strokes are ischemic in origin (3), limited data exist comparing anterior circulation (AC) and posterior circulation (PC) AIS. A deeper understanding of the distinct characteristics that differentiate AC from PC strokes is essential for advancing personalized therapeutic approaches in stroke medicine (4, 5). The intrinsic heterogeneity in stroke presentation, risk profiles, and clinical outcomes underscores the need to move beyond a “one-size-fits-all” paradigm toward a more nuanced understanding of these subtypes. Such refinement forms the foundation for developing targeted therapeutic strategies, enhancing clinical outcomes, and promoting more efficient allocation of healthcare resources (6, 7). An often underrecognized yet clinically relevant aspect of stroke epidemiology lies in the distribution patterns of cerebral circulation events. Although the posterior circulation receives only approximately 20% of total cerebral blood flow, it accounts for 20%–25% of all ischemic strokes (8–10).

This disproportion suggests unique vulnerability factors and underlying pathophysiological mechanisms that remain insufficiently understood and require further investigation (11). Clarifying these distinctions is not merely academic—it is essential for optimizing risk stratification and guiding the development of subtype-specific preventive and therapeutic strategies. Notably, prior studies have reported meaningful differences between AC and PC strokes in terms of vascular risk profiles and responses to acute interventions, reinforcing the need for tailored clinical approaches (12, 13).

Growing evidence demonstrates marked differences in patient demographics, cardiovascular risk factors, and stroke etiology between anterior and posterior circulation strokes (5). These differences extend beyond anatomical distribution and encompass a broad range of clinical presentations, risk profiles, and biological markers. However, inconsistencies across studies underscore the need for a more rigorous and comprehensive investigation to reconcile conflicting findings and establish a robust evidence base to inform clinical practice. Such systematic efforts are particularly critical as healthcare systems increasingly adopt precision medicine frameworks, aiming to align therapeutic strategies with individual patient characteristics and stroke subtype– specific features. A critical challenge in this paradigm is the accurate assessment of stroke severity in posterior circulation (PC) strokes. The widely used National Institutes of Health Stroke Scale (NIHSS) (14) has demonstrated substantial limitations in evaluating PC strokes (14–17), with PC strokes consistently assigned lower scores despite significant neurological deficits. These findings highlight the scale’s limited sensitivity to hallmark PC symptoms—such as truncal ataxia, nystagmus, cranial nerve palsies, and dysphagia—thereby increasing the risk of under-recognition and inappropriate clinical decisions. This measurement bias may lead to inadvertent exclusion from acute reperfusion therapies such as thrombolysis and thrombectomy, and may also compromise outcome prediction and rehabilitation strategies. Inflammatory markers—particularly C-reactive protein (CRP) and white blood cell (WBC) counts—have emerged as focal points in stroke research, offering promising avenues for improving risk stratification, prognostication, and the development of targeted therapies (18). C-reactive protein, a well-established indicator of systemic inflammation, has been shown to rise following cerebral ischemia (19, 20). Despite its clinical utility, direct comparisons of CRP levels between anterior (AC) and posterior (PC) circulation strokes remain scarce, leaving a critical gap in our understanding of stroke subtype–specific inflammatory responses. Kim et al. (21) assessed CRP levels exclusively in PC stroke patients, demonstrating an association with stroke severity but without including AC cases for comparison. In one of the few studies conducting a direct comparison, De Marchis et al. (11) reported a non-significant trend toward higher CRP levels in AC strokes (*p* = 0.06). A similar knowledge gap applies to white blood cell counts. Although limited, available data suggest that PC stroke patients—particularly those with large vessel occlusions—may present with higher total WBC and neutrophil levels compared to those with AC strokes (22). Such differences in inflammatory profiles may contribute to variations in treatment response and long-term outcomes, highlighting the potential of biomarker-informed therapeutic strategies tailored to stroke location and underlying pathophysiology. In parallel, the relationship between atrial fibrillation (AF)—a major modifiable stroke risk factor—and stroke subtype warrants closer examination. AF is a well-established independent risk factor for both embolic and non-embolic strokes (23), with reported prevalence rates among ischemic stroke patients ranging from 15% to 38% (24). However, its prevalence appears to differ significantly between AC and PC strokes. For example, Subramanian et al. (25) reported a markedly lower frequency of AF in PC stroke patients, suggesting the involvement of distinct embolic mechanisms or protective features such as enhanced collateral circulation. These differences may influence key clinical decisions, including anticoagulation strategies and secondary prevention protocols. Further investigation is needed to clarify the biological underpinnings and clinical implications of this observed divergence.

Age is a well-recognized demographic factor in stroke risk and outcomes; however, its role in differentiating anterior (AC) and posterior circulation (PC) strokes remains inconsistent across the literature. Early studies suggested a tendency for PC strokes to occur in younger patients Puustjärvi et al. (26); Libman et al. (27), while Tao et al. (4) reported statistically significant age differences between AC and PC strokes. More recent findings indicate a more complex relationship. Kim et al. (28) demonstrated that vertebral artery origin stenosis (VAO) constitutes a significant risk factor for PC stroke recurrence, particularly in patients with underlying vascular pathology. Although age itself was not identified as an independent predictor, older patients with PC strokes were more likely to exhibit progressive atherosclerotic changes, including VAO—suggesting an age-related vulnerability in the vertebrobasilar system and supporting the need for age-adjusted risk stratification. [Frame3]Gender differences in stroke risk and anatomical distribution have increasingly been recognized as a significant area of investigation, with important implications for both primary prevention and the customization of clinical management strategies (29). Several studies have reported a higher proportion of males among patients with posterior circulation (PC) strokes (4, 26). However, the underlying mechanisms driving this observed difference remain incompletely understood and warrant further exploration. Mehndiratta et al. (30) proposed that referral bias—namely, differences in how men and women with stroke symptoms are evaluated and referred for care— may contribute to these gender differences. Additionally, Perko et al. (31) identified notable variations in cerebrovascular reactivity between anterior and posterior circulation territories, which may help explain sex-related differences in stroke susceptibility and treatment response, suggesting a potential biological basis for the observed variation.

Our group’s recent investigations have offered additional insights into the complex interplay of factors influencing stroke outcomes, extending beyond conventional vascular risk profiles. We demonstrated that vagal nerve activity, a physiological marker of autonomic nervous system function, exerts a meaningful moderating effect on stroke outcomes, underscoring its relevance in prognostic modeling and rehabilitation planning (32). In parallel, our examination of ethnic and gender variations in ischemic stroke patterns revealed significant differences in stroke characteristics across diverse demographic groups, highlighting the necessity of incorporating demographic and cultural variables into stroke research (33). Collectively, these findings reinforce the imperative for personalized approaches that are sensitive to the unique physiological, demographic, and cultural characteristics of each patient. The primary objective of this study is to conduct a comprehensive investigation into the multifaceted differences in risk factors, stroke severity, and clinical outcomes between anterior (AC) and posterior circulation (PC) strokes. Particular emphasis is placed on identifying distinct and clinically meaningful signatures—demographic, clinical, and biological (specifically, inflammatory markers)—that may inform the development and implementation of personalized therapeutic strategies in stroke care. By systematically examining multiple dimensions of stroke presentation—from inflammatory profiles to clinical manifestations and functional outcomes—this study aims to establish a robust, evidence-based foundation for advancing individualized approaches to stroke prevention, acute assessment, management, and rehabilitation. The present work moves beyond broad generalizations, seeking instead to characterize the specific attributes of each stroke subtype and thereby contribute to the evolution of precision stroke care—where treatment decisions are guided by patient-specific and subtype-specific profiles rather than a one-size-fits-all model.

## 2 Materials and methods

This retrospective, observational-analytical study examined the medical records of patients diagnosed with ischemic stroke at the Galilee Medical Center, a tertiary care center and teaching hospital in Northern Israel, between March 2023 and February 2025. The study population comprised 499 patients, categorized into two groups based on stroke location: anterior circulation (AC) (*n* = 434) and posterior circulation (PC) (*n* = 65).

### 2.1 Inclusion and exclusion criteria

The study included patients aged 18 years or older with a confirmed diagnosis of ischemic stroke, based on clinical presentation and neuroimaging findings [computed tomography (CT) or magnetic resonance imaging (MRI)]. To maintain a focus on the distinct characteristics of AC and PC strokes and to ensure homogeneity within the study groups, patients with lacunar strokes were excluded, as were cases of Transient Ischemic Attack (TIA) and ischemic strokes with mild occlusion (i.e., only cases with moderate or greater vessel occlusion were included). This exclusion criterion was implemented to minimize confounding factors associated with different stroke mechanisms, thereby enhancing the validity and reliability of the comparisons between AC and PC stroke subtypes. Following approval from the local Hospital Ethics Committee, the study cohort was obtained by requesting the Medical Records Department to utilize their automated systems for extracting patient files admitted during the study period (March 2023–February 2025). This extraction was strictly based on pre-defined diagnostic codes that matched the specific inclusion criteria: (1) Ischemic stroke (non-lacunar); (2) occlusion of moderate or greater severity; and (3) exclusion of hemorrhagic stroke and Transient Ischemic Attack (TIA). This automated process ensured that the data received was pre-filtered, and the initial exclusion of non-relevant stroke types occurred at the computerized source. Patients with lacunar strokes—defined as small, deep infarcts less than 15 mm in maximum diameter on neuroimaging were subject to a secondary, manual review by the study team to confirm strict adherence to the inclusion criteria. This rigorous selection process yielded a final analytic cohort of 499 patients: 434 with anterior circulation (AC) strokes and 65 with posterior circulation (PC) strokes.

### 2.2 Variables and data collection

Comprehensive data collection was performed, adhering to strict protocols to ensure data quality and consistency. Stroke location (AC vs. PC) served as the primary outcome variable. Stroke location was determined based on neuroimaging findings (CT and/or MRI) and established clinical criteria, with adjudication by a senior neurologist in cases of ambiguity. Anterior circulation (AC) strokes were defined as ischemic lesions located in the vascular territories supplied by the internal carotid arteries and their branches, specifically the anterior cerebral artery (ACA) and middle cerebral artery (MCA). Posterior circulation (PC) strokes were defined as ischemic lesions in the territories supplied by the vertebral arteries, basilar artery, and posterior cerebral arteries (PCA), including structures such as the brainstem, cerebellum, thalamus, and occipital lobes. The classification was based on the anatomical location of the infarct as visualized on neuroimaging, correlated with established vascular territory maps. For patients who underwent both CT and MRI during their hospital stay, the final stroke classification was determined using a standardized hierarchical approach: MRI with diffusion-weighted imaging (DWI) was prioritized when available, given its superior sensitivity for detecting acute ischemic changes, particularly in the posterior fossa where CT has known limitations due to beam-hardening artifacts. In cases where initial non-contrast CT was negative or inconclusive but clinical suspicion for stroke remained high based on neurological examination, MRI was performed for definitive diagnosis. The integration of findings from both modalities followed a predefined protocol: first, non-contrast CT was used for rapid initial assessment and exclusion of hemorrhage; second, MRI with DWI sequences was obtained when feasible, especially for suspected PC strokes or when CT findings were equivocal; and third, the final vascular territory assignment was determined by correlating the anatomical distribution of the ischemic lesion on the most definitive imaging study with standard arterial territory atlases and clinical presentation. All neuroimaging studies were reviewed and classified independently by a board-certified neuroradiologist with expertise in cerebrovascular imaging, in consultation with the treating neurologist. In cases of diagnostic uncertainty or discordance between imaging findings and clinical presentation, a consensus decision was reached through multidisciplinary discussion. Demographic information, including age, gender, and ethnicity (Jewish, Arab, or other), was recorded for each patient. The study assessed a comprehensive panel of vascular risk factors. Smoking status was categorized as current smoker, former smoker, or never smoker. Diabetes mellitus was defined by a prior diagnosis of diabetes, current use of glucose-lowering medications, or HbA1c levels ≥ 6.5% at the time of admission. Hypertension was defined by a prior diagnosis of hypertension, current use of antihypertensive medications, or blood pressure measurements ≥ 140/90 mmHg on repeated measurements during the hospital stay. Hyperlipidemia was defined by a prior diagnosis of hyperlipidemia, current use of lipid-lowering medications, or lipid profile abnormalities (total cholesterol, LDL cholesterol, HDL cholesterol, and triglycerides) based on established clinical guidelines. Ischemic heart disease was defined by a prior history of myocardial infarction, angina pectoris, or coronary revascularization procedures (percutaneous coronary intervention or coronary artery bypass grafting). Previous stroke history was defined by a documented history of prior ischemic or hemorrhagic stroke. Atrial fibrillation (AF) was documented by electrocardiogram (ECG) findings during the hospital stay or a prior diagnosis of AF. Clinical and laboratory parameters were systematically collected for each patient upon admission to the hospital. These included the baseline NIHSS score, a standardized assessment of stroke severity, encompassing level of consciousness, visual fields, motor function, sensory function, language, and neglect performed by trained neurologists or stroke nurses. Patient weight was measured in kilograms (kg). Coagulation parameters, including prothrombin time (PT), partial thromboplastin time (PTT), and international normalized ratio (INR), were measured for all patients, with particular attention to INR values for patients receiving anticoagulant therapy. These parameters were assessed to evaluate the coagulation status of patients at baseline. Blood samples for all laboratory parameters, including inflammatory markers, were drawn upon patient admission to the emergency department, typically within the first few hours of arrival and prior to the initiation of specific stroke therapies. This initial sample reflects the patient’s inflammatory state at presentation. White blood cell (WBC) counts (×10^9^/L) were determined using an automated hematology analyzer via flow cytometry. C-reactive protein (CRP) levels (mg/L) were quantified using a high-sensitivity particle-enhanced immunoturbidimetric assay on an automated clinical chemistry analyzer. These markers were selected as indicators of systemic inflammation, which has been implicated in stroke pathogenesis and outcomes. All analyses were performed at the hospital’s accredited central laboratory according to standard operating procedures. Length of hospital stay was measured in days, from the date of admission to the date of discharge. Discharge destination was categorized as home, rehabilitation facility, skilled nursing facility, or other.

### 2.3 Statistical analysis

**FIGURE 1:**
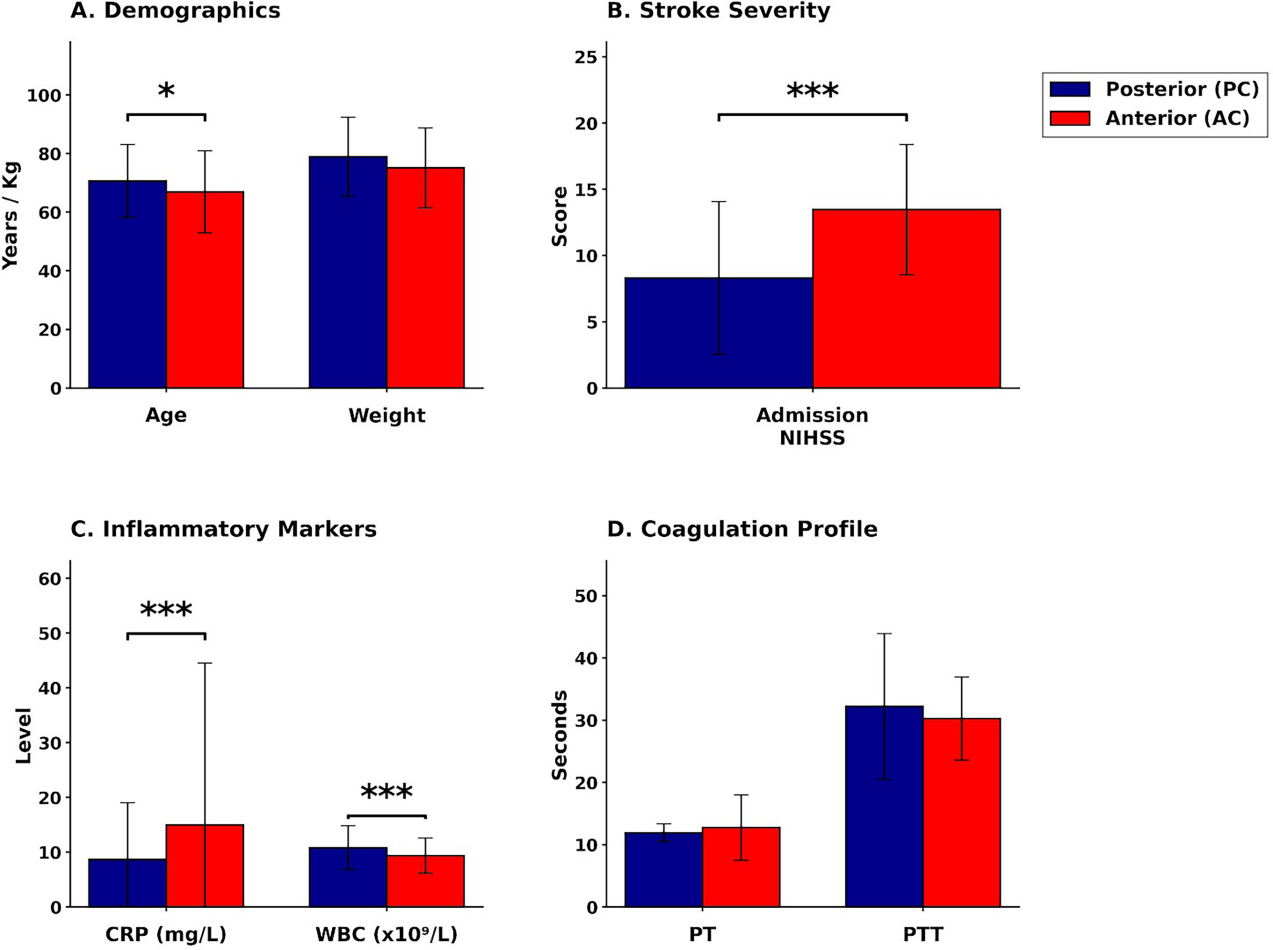
Comparison of clinical and laboratory variables between anterior and posterior circulation stroke groups. The figure displays raw mean values ± standard deviation (SD). Blue bars represent posterior circulation (PC) strokes; red bars represent anterior circulation (AC) strokes. **(A)** Demographics: Patients with PC strokes were significantly older (*p < 0.05). **(B)** Stroke severity: Admission NIHSS scores were significantly higher in AC strokes (***p < 0.001). Higher scores indicate greater neurological impairment. **(C)** Inflammatory markers: CRP levels were markedly elevated in AC strokes, whereas WBC counts were significantly higher in PC strokes (***p < 0.001 for both). **(D)** Coagulation: No significant differences were found in PT or PTT. NIHSS, National Institutes of Health Stroke Scale; CRP, C-reactive protein; WBC, white blood cell count; PT, prothrombin time; PTT, partial thromboplastin time. Significance: *p < 0.05, ***p < 0.001, ns, non-significant.

All statistical analyses were performed using IBM SPSS Statistics for Windows, version 28.0 (IBM Corporation, Armonk, NY, USA). Descriptive statistics were calculated for all study variables. Continuous variables were expressed as mean ± standard deviation, and categorical variables were expressed as frequencies and percentages. Comparisons of continuous variables [age, NIHSS scores at baseline and discharge, weight, coagulation parameters including prothrombin time (PT), partial thromboplastin time (PTT), and international normalized ratio (INR), C-reactive protein (CRP), white blood cell count (WBC), and length of hospital stay] between anterior circulation (AC) and posterior circulation (PC) stroke groups were conducted using independent samples *t*-tests. The results are presented in a multipanel bar graph (Figure 1). Comparisons of categorical variables (gender, nationality, smoking status, previous stroke history, hyperlipidemia, diabetes mellitus, and atrial fibrillation) between the two groups were performed using Pearson’s chi-square test. A two-tailed *p*-value of less than 0.05 was considered statistically significant for all comparisons. To identify independent predictors of stroke location, two separate multivariable binary logistic regression analyses were performed. The dependent variable in both models was stroke location, coded as a binary outcome (anterior circulation versus posterior circulation). The first model included baseline demographic and clinical predictor variables: age (years, continuous), baseline NIHSS score (continuous), gender (male versus female, categorical), atrial fibrillation (present versus absent, categorical), and previous stroke history (yes versus no, categorical). The second model included outcome-related predictor variables: white blood cell count (×10^9^/L, continuous), C-reactive protein level (mg/L, continuous), length of hospital stay (days, continuous), and NIHSS score at discharge (continuous). Predictor variables were selected based on clinical relevance to stroke subtypes as identified in prior literature and their potential pathophysiological relationship to stroke location. This selection process followed a two-stage, theory-driven consistency standard: variables were first screened based on a univariate trend (*p* < 0.2), and final inclusion was determined by preliminary comparative model testing to optimize overall predictive performance (e.g., maximizing Nagelkerke and classification accuracy), while prioritizing variables with established clinical necessity and theoretical importance. All predictor variables were entered simultaneously into each model using the Enter method. For each logistic regression model, the following statistics were calculated and reported: unstandardized regression coefficients (B), standard errors (SE), odds ratios [Exp(B) or exponentiated B coefficients], 95% confidence intervals (CI) for the odds ratios, and *p*-values for individual predictors. Model fit was evaluated using the Nagelkerke R-squared statistic, which estimates the proportion of variance in the dependent variable explained by the predictor variables. Overall classification accuracy was calculated as the percentage of cases correctly classified by the model. Statistical significance for individual predictors was assessed using the Wald chi-square test, with *p*-values less than 0.05 considered statistically significant. The Hosmer–Lemeshow test was used to assess model calibration. All statistical tests were two-tailed, and *p*-values less than 0.05 were considered statistically significant throughout the study. No adjustments for multiple comparisons were applied, as the analyses were hypothesis-driven rather than exploratory.

## 3 Results

### 3.1 Cohort characteristics

The baseline characteristics and cardiovascular risk factors for the entire study cohort are summarized in Table 1. The cohort comprised 499 ischemic stroke patients, with 434 (87.0%) classified as anterior circulation (AC) stroke and 65 (13.0%) classified as posterior circulation (PC) stroke.

**TABLE 1:** Comparison of baseline demographic characteristics and cardiovascular risk factors in anterior and posterior circulation stroke groups.

Characteristic	Posterior circulation (PC) (*n* = 65)	Anterior circulation (AC) (*n* = 434)	*p*-value
**Continuous variables (mean ± SD)**			
Age (years)	70.65 ± 12.48	66.88 ± 13.99	< 0.05*
Weight (kg)	78.88 ± 13.57	75.12 ± 13.56	0.304
**Categorical variables [*n* (%)]**			
Male gender	45 (69.2%)	221 (50.9%)	< 0.007**
Nationality (Arab)	29 (44.6%)	181 (41.7%)	0.662
Current smoker	20 (30.8%)	104 (24.0%)	0.237
Previous stroke history	13 (20.0%)	45 (10.4%)	< 0.024*
Hyperlipidemia	37 (56.9%)	278 (64.2%)	0.256
Diabetes mellitus	25 (38.5%)	167 (38.5%)	0.995
Atrial fibrillation	15 (23.1%)	151 (34.9%)	< 0.044*

*p < 0.05, **p < 0.01. Continuous variables are presented as mean ± standard deviation (SD) and compared using independent samples *t*-tests. Categorical variables are presented as *n* (%) and compared using Pearson’s chi-square tests.

### 3.2 Differences in risk factors and inflammatory markers between anterior and posterior stroke groups

Significant differences in age, baseline NIHSS, and inflammatory markers were observed between the anterior and posterior stroke groups (Table 2 and Figure 1). Patients with posterior circulation (PC) stroke were significantly older (Mean = 70.65, SD = 12.48 years) compared to those with anterior circulation (AC) stroke (Mean = 66.88, SD = 13.99 years; *p* < 0.05). Baseline NIHSS scores, reflecting initial stroke severity, were significantly higher in the AC group (Mean = 13.47, SD = 4.92) than in the PC group (Mean = 8.31, SD = 5.76; *p* < 0.001). Furthermore, distinct inflammatory profiles were evident: C-reactive protein (CRP) levels were significantly higher in AC strokes, while white blood cell (WBC) counts were significantly higher in PC strokes (Figure 1). Categorical risk factors were also compared between the AC and PC stroke groups (Table 1). A significantly higher proportion of males was observed in the PC group (69.2%) compared to the AC group (50.9%; *p* = 0.007). Furthermore, a history of previous stroke was significantly more frequent in patients with PC strokes (20%) than in those with AC strokes (10.4%; *p* = 0.024). Conversely, the prevalence of atrial fibrillation was significantly lower in the PC group (23.1%) compared to the AC group (34.9%; *p* = 0.044). No statistically significant differences were found between the two groups in terms of nationality, smoking status, hyperlipidemia, or diabetes prevalence.

**TABLE 2:** Comparison of continuous risk factors and inflammatory markers between anterior and posterior stroke groups.

Variables	Posterior (*n* = 65)	Anterior (*n* = 434)	*p*-value
Age (years)	70.65 ± 12.48	66.88 ± 13.99	< 0.05*
NIHSS at baseline	8.31 ± 5.76	13.47 ± 4.92	< 0.001***
Weight (kg)	78.88 ± 13.57	75.12 ± 13.56	0.304
PT (seconds)	11.92 ± 1.44	12.74 ± 5.24	0.230
PTT (seconds)	32.23 ± 11.71	30.26 ± 6.68	0.059
INR	1.04 ± 0.15	1.06 ± 0.14	0.561
CRP (mg/L)	8.65 ± 10.37	14.97 ± 29.53	< 0.001***
WBC (×10^9^/L)	10.80 ± 4.01	9.36 ± 3.22	< 0.001***

Data presented as mean ± standard deviation. *p < 0.05, ***p < 0.001.

### 3.3 Prediction of stroke location based on risk factors

To identify independent predictors of stroke location, a multivariable logistic regression model was constructed (Table 3). The model, incorporating age, baseline NIHSS score, gender, atrial fibrillation, and previous stroke history as potential predictors, explained 10.6% of the variance in stroke location (Nagelkerke R Square = 0.106) and demonstrated an overall classification accuracy of 87.7%. Baseline NIHSS score emerged as a highly significant predictor of stroke location (OR = 1.20, 95% CI: 1.13–1.27, *p* < 0.001), with higher scores strongly associated with an increased likelihood of AC stroke. Gender was also identified as a significant predictor (OR = 0.984, 95% CI: 0.97–1.00, *p* = 0.050), suggesting that male gender was associated with a decreased likelihood of AC stroke. However, age, atrial fibrillation, and previous stroke history did not reach statistical significance as independent predictors in this model.

**TABLE 3:** Logistic regression analysis predicting stroke location based on risk factors.

Variable	B	SE	Exp (B)	95% CI	*p*-value
Age	0.001	0.012	1.01	0.98–1.02	0.9
NIHSS at baseline	0.18	0.03	1.2	1.13–1.27	< 0.001**
Atrial fibrillation	0.35	0.344	1.42	0.73–2.77	0.3
Gender (male)	−0.59	0.307	0.984	0.97–1.00	0.05*
Previous stroke	0.48	0.38	1.62	0.75–3.45	0.207

*p < 0.05, ***p < 0.001. SE, standard error; Exp(B), odds ratio; CI, confidence interval.

### 3.4 Differences in outcome measures between anterior and posterior stroke groups

Significant differences were observed between the AC and PC stroke groups in terms of NIHSS scores at discharge, CRP levels, and WBC counts (Table 4). The AC group exhibited significantly higher NIHSS scores at discharge (Mean = 6.00, SD = 5.12) compared to the PC group (Mean = 4.09, SD = 4.98; *p* = 0.004). CRP levels were also significantly higher in the AC group (Mean = 14.97, SD = 29.53) than in the PC group (Mean = 8.65, SD = 10.37; *p* < 0.001). Conversely, WBC counts were significantly higher in the PC group (Mean = 10.80, SD = 4.01) compared to the AC group (Mean = 9.36, SD = 3.22; *p* < 0.001). No statistically significant differences were found between the two groups in terms of prothrombin time (PT) or length of hospital stay.

**TABLE 4:** Comparison of outcome measures between anterior and posterior stroke groups.

Variable	Posterior (*n* = 65)	Anterior (*n* = 434)	*p*-value
NIHSS at discharge	4.09 ± 4.98	6.00 ± 5.12	< 0.01**
CRP (mg/L)	8.65 ± 10.37	14.97 ± 29.53	< 0.001***
WBC (×10^9^/L)	10.80 ± 4.01	9.36 ± 3.22	< 0.001***
PT (seconds)	11.92 ± 1.44	12.74 ± 5.24	0.113
Length of stay (days)	15.06 ± 14.28	13.33 ± 16.80	0.215

Data presented as mean ± standard deviation. **p < 0.01, *** *p* < 0.001.

### 3.5 Prediction of stroke location based on outcome measures

A second logistic regression model was constructed to predict stroke location using outcome variables as predictors (Table 5). This model explained 10.4% of the variance in stroke location (Nagelkerke R Square = 0.104) and achieved an overall classification accuracy of 87.6%. WBC count (OR = 0.871, 95% CI: 0.79–0.96, *p* = 0.003), length of hospital stay (OR = 0.960, 95% CI: 0.930.99, *p* = 0.010), and NIHSS score at discharge (OR = 1.157, 95% CI: 1.06–1.27, *p* < 0.01) emerged as significant independent predictors of stroke location.

**TABLE 5:** Logistic regression analysis predicting stroke location based on outcome measures.

Variable	B	SE	Exp(B)	95% CI	*p*-value
WBC (×10^9^/L)	−0.138	0.047	0.871	0.79–0.96	< 0.01**
CRP (mg/L)	0.015	0.009	1.015	0.99–1.03	0.105
Length of stay (days)	−0.041	0.016	0.96	0.93–0.99	< 0.05*
NIHSS at discharge	0.146	0.046	1.157	1.06–1.27	< 0.01**

*p < 0.05, **p < 0.01. SE, standard error; Exp(B), odds ratio; CI, confidence interval.

Higher WBC counts and longer hospital stays were associated with a decreased likelihood of AC stroke, while higher NIHSS scores at discharge were associated with an increased likelihood of AC stroke. CRP levels did not reach statistical significance as an independent predictor in this model.

To provide a comprehensive overview, Table 6 presents a summary of the variables significantly associated with posterior circulation stroke.

**TABLE 6:** Summary of factors independently associated with posterior circulation stroke in multivariable logistic regression analysis.

Variable	Odds ratio (95% CI)	*p*-value
Baseline NIHSS	0.833 (0.79–0.88)	< 0.001***
Male gender	1.016 (1.00–1.03)	< 0.05*
WBC count (×10^9^/L)	1.148 (1.04–1.26)	< 0.01**
Length of hospital stay	1.042 (1.01–1.08)	< 0.05*
NIHSS score at discharge	0.864 (0.78–0.96)	< 0.01**

*p < 0.05, **p < 0.01, ***p < 0.001.

## 4 Discussion

This comprehensive investigation into the distinctions between anterior (AC) and posterior circulation (PC) strokes highlights key differences with potential implications for advancing more personalized approaches in stroke care. The findings suggest that AC and PC strokes may represent pathophysiological distinct entities, each characterized by unique constellations of risk factors, inflammatory signatures, and clinical manifestations that could benefit from tailored therapeutic strategies. The observed interplay among demographic characteristics, inflammatory markers, and clinical outcomes challenges the adequacy of uniform assessment and treatment paradigms. These results, while derived from a single-center retrospective cohort, add to a growing recognition of stroke heterogeneity and underscore the need for continued investigation into circulation-specific mechanisms. While further research is warranted to validate these findings and elucidate underlying pathophysiological processes, the present data underscore the relevance of adopting more individualized models of stroke care—spanning from initial evaluation through acute management to long-term rehabilitation and secondary prevention.

### 4.1 Age and gender differences: clinical and pathophysiological implications

Our analysis identified distinct demographic patterns that may carry important implications for individualized risk stratification and targeted prevention strategies. Patients with posterior circulation (PC) strokes were significantly older than those with anterior circulation (AC) strokes (mean age 70.65 vs. 66.88 years, *p* < 0.05). This finding contrasts with earlier studies that reported a higher prevalence of PC strokes among younger individuals (26), yet is more consistent with the findings of Kim et al. (28), who observed an increased prevalence of vertebral artery origin stenosis—a recognized risk factor for PC ischemia—in older patients. This age-related pattern may reflect the cumulative burden of vascular risk factors over time, contributing to progressive atherosclerosis in the vertebrobasilar system and increased susceptibility to ischemic events. These findings underscore the importance of age-adjusted risk assessment models and support the need for more intensive vascular risk management among older adults at risk for PC stroke. With respect to sex-based differences, our findings revealed a significantly higher proportion of males among PC stroke patients compared to the AC group (69.2% vs. 50.9%, *p* = 0.007). This observation is consistent with the findings of Zürcher et al. (5), who similarly reported a higher proportion of males in the PC stroke subgroup relative to the AC group. However, other studies have yielded mixed results, with some failing to identify significant sex-related differences in stroke subtype prevalence or clinical presentation (27, 34).

Although the basis for this sex-related distribution is not fully understood, several explanatory models have been proposed. One hypothesis points to referral bias, whereby men and women may be evaluated or referred differently when presenting with stroke symptoms, as suggested by Mehndiratta et al. (30). Alternatively, biological mechanisms may be implicated: Perko et al. (31) identified variations in cerebrovascular reactivity to L-arginine between the anterior and posterior circulation, potentially explaining gender-based differences in stroke risk due to differences in hormonal influences or vascular anatomy. These findings suggest that the relationship between gender and stroke location may be influenced by a complex interplay of hormonal factors, vascular anatomy, differences in the prevalence of specific risk factors (such as diabetes, as suggested by Subramanian et al. (25) —as well as differences in healthcare-seeking behavior and access to care). Together, these factors may help account for the observed male predominance in PC stroke.

### 4.2 Stroke severity and clinical manifestations: implications for assessment and prognosis

Our study identified critical differences in stroke severity between anterior circulation (AC) and posterior circulation (PC) strokes, which call into question the adequacy of current assessment paradigms and carry meaningful implications for clinical decision-making and prognostication. The significantly lower baseline NIHSS scores observed in the PC group compared to the AC group (mean 8.31 vs. 13.47, *p* < 0.001) constitute a clinically relevant finding that warrants careful interpretation. As highlighted by Zeng et al. (35), this discrepancy likely reflects inherent limitations of the NIHSS in capturing the full range of neurological deficits characteristic of PC strokes. Although the NIHSS remains the most widely used tool for quantifying stroke severity, its design prioritizes deficits more common in AC strokes—such as aphasia and hemiparesis— while underrepresenting hallmark features of PC strokes, including truncal ataxia, nystagmus, cranial nerve involvement, and dysphagia (36). As a result, patients with PC strokes may be systematically assigned lower severity scores, leading to potential under-recognition and subsequent delays or exclusions from appropriate acute interventions such as intravenous thrombolysis or mechanical thrombectomy. This underestimation, as discussed by Guenego et al. (37), may profoundly influence clinical decision-making, contribute to suboptimal prognostic assessments, and impact the allocation of rehabilitation resources—ultimately compromising longterm patient outcomes. Importantly, the observed differences in severity extended beyond initial presentation. NIHSS scores at discharge remained significantly higher in the AC group compared to the PC group (mean 6.00 vs. 4.09, *p* = 0.004), suggesting that standard outcome measures may not adequately reflect the true burden of impairment in PC stroke.

This persistent discrepancy underscores the complex interplay between stroke location, neurological symptomatology, and the limitations of current assessment tools in capturing the full clinical trajectory. While a lower initial NIHSS score in PC stroke might appear indicative of milder disease, emerging evidence suggests otherwise. Inoa et al. (16) demonstrated that patients with PC strokes can present with substantial neurological deficits that are underrepresented by the NIHSS, leading to potential misclassification of stroke severity. Furthermore, Alemseged et al. (17) proposed a posterior-specific adaptation of the NIHSS, which significantly improved the accuracy of prognostic predictions in PC stroke, emphasizing the need for subtype-sensitive assessment tools. Alexandre et al. (36) similarly highlighted that patients with posterior large-vessel occlusions may exhibit deceptively low NIHSS scores despite clinically significant impairment. Collectively, these findings support the need for more sensitive and comprehensive instruments capable of capturing the distinct clinical features often seen in PC strokes—such as truncal ataxia, cranial nerve dysfunction, and dysphagia—that are frequently overlooked by the standard NIHSS. Developing such tools is essential for ensuring accurate severity assessment, guiding timely interventions, and informing individualized rehabilitation strategies.

### 4.3 Inflammatory markers and biological signatures: unveiling distinct pathophysiological mechanisms

A key novel finding of this study is the identification of a divergent inflammatory signature between stroke subtypes. While prior research has offered fragmented insights, our work is among the first to demonstrate a clear, opposing pattern within a single cohort. The observation of significantly higher C-reactive protein (CRP) levels in the AC group (Mean = 14.97 vs. 8.65 mg/L, *p* < 0.001) aligns with a non-significant trend previously reported by De Marchis et al. (11) and is consistent with findings by Elbelkimy et al. (20) and Yu et al. (19). This CRP-dominant response in AC strokes may stem from factors such as larger infarct volumes, a higher prevalence of atherosclerotic disease (particularly involving the carotid arteries), and a more pronounced systemic inflammatory response reflecting the extent and severity of cerebral ischemia (38). The tendency of AC strokes to involve larger vessel occlusions affecting broader cerebral territories (39), likely contributes to greater release of inflammatory mediators, thereby amplifying systemic CRP levels.

In contrast, and completing this divergent pattern, the significantly elevated white blood cell (WBC) counts observed in PC strokes (Mean = 10.80 vs. 9.36 × 10^9^/L, *p* < 0.001) are in line with findings from Tarkanyi et al. (22), who suggested higher leukocyte levels in PC strokes with large vessel occlusions. This leukocyte-dominant response, occurring despite lower clinical severity scores, may reflect a distinct inflammatory trajectory, possibly related to variations in the microvascular structure of the posterior circulation or differential permeability of the blood-brain barrier (40). Alternatively, it may reflect a greater burden of chronic inflammatory conditions, such as those associated with diabetes and hypertension, which were prevalent in this subgroup (41).

Taken together, these contrasting inflammatory profiles—a CRP-dominant response in AC strokes and a leukocyte dominant response in PC strokes—highlight fundamental differences in the biological response to ischemic injury across stroke subtypes. This divergence may have important clinical implications, particularly in guiding the development of inflammation-targeted therapeutic strategies. Although further research is required to elucidate the precise mechanisms involved, these findings underscore the potential relevance of biomarker-guided, subtype-specific anti-inflammatory approaches in stroke care.

### 4.4 Risk factors and comorbidity patterns: implications for prevention

The analysis of traditional vascular risk factors revealed significant and clinically relevant differences in their distribution between anterior circulation (AC) and posterior circulation (PC) stroke subtypes, particularly regarding atrial fibrillation (AF) and prior stroke history. The significantly lower prevalence of AF in the PC stroke group (23.1% vs. 34.9%, *p* = 0.044) is consistent with previous findings suggesting that distinct pathophysiological mechanisms may underlie PC stroke etiology.

While AF is a well-established major risk factor for cardioembolic stroke—associated with increased mortality, disability, and hospitalization (24, 42, 43) —its lower prevalence in PC strokes suggests that alternative mechanisms, such as *in situ* thrombosis or vertebrobasilar atherosclerosis, might play a more prominent role (25). This distinction has important implications for risk stratification and antithrombotic therapy decisions. Patients with PC strokes without documented AF may not benefit from anticoagulation to the same extent as patients with AF associated AC strokes, highlighting the potential preference for alternative antiplatelet strategies or interventions targeting vertebrobasilar pathology. A recent study focusing on patients with vertebrobasilar dolichoectasia—an anatomical variant involving arterial elongation and dilation in the posterior circulation—found a significantly increased risk of recurrent stroke associated with larger basilar artery diameter and diffuse intracranial dolichoectasia. Although the study did not specifically exclude patients with AF, its findings support the relevance of local vascular mechanisms as contributors to recurrence risk in certain PC stroke populations (44).

The significantly higher rate of previous stroke observed in the PC stroke group (20% vs. 10.4%, *p* = 0.024) raises important questions about secondary prevention strategies and highlights potential differences in recurrence risk patterns between stroke subtypes. This finding suggests that patients with PC strokes may represent a higher-risk population for recurrent events, potentially due to differences in underlying vascular disease, reduced responsiveness to current secondary prevention strategies, or other yet-unidentified factors. This observation underscores and reinforces the need for more aggressive secondary prevention measures in patients with PC strokes, including optimized medical management, targeted lifestyle modifications, and potentially more intensive monitoring for recurrent events. Further research is needed. Additional studies are required to identify the specific factors contributing to this increased recurrence risk and to develop tailored secondary prevention strategies for patients with PC stroke.

### 4.5 Clinical implications and the path toward precision medicine in stroke care

The substantial evidence presented in this study—demonstrating marked differences between AC and PC strokes across multiple domains including demographics, clinical presentation, inflammatory markers, and risk factor profiles—highlights the pressing need for a shift in stroke care paradigms. The conventional “one-size-fits-all” model, which largely overlooks these fundamental distinctions, appears increasingly insufficient in light of growing recognition of stroke subtype heterogeneity.

The consistently lower NIHSS scores observed in PC strokes (Mean = 8.31 vs. 13.47, *p* < 0.001), despite clinical evidence of severe neurological deficits often not captured by the NIHSS, underscore a critical limitation in current assessment tools. This pattern, well-documented in previous studies (4, 5, 7, 11), suggests that relying exclusively on NIHSS may lead to systematic underestimation of PC stroke severity. Such misclassification may, in turn, result in delays in diagnosis, suboptimal triage, and inappropriate therapeutic decisions—ultimately compromising clinical outcomes. These findings reinforce the need for the development and validation of stroke subtype–specific assessment tools that accurately capture the unique clinical manifestations of PC strokes, such as vertigo, ataxia, diplopia, and cranial nerve involvement—features frequently underrepresented in standard assessments.

Additionally, the distinct inflammatory profiles identified in this study—significantly higher CRP levels in AC strokes (Mean = 14.97 vs. 8.65 mg/L, *p* < 0.001) and elevated WBC counts in PC strokes (Mean = 10.80 vs. 9.36 × 10^9^/L, *p* < 0.001)—provide strong support for differentiated therapeutic approaches based on stroke subtype. These biological distinctions, corroborated by prior research (3, 8, 9, 11, 24, 25, 28–31), suggest that inflammatory responses to cerebral ischemia may vary by vascular territory. In AC strokes, elevated CRP may reflect a heightened systemic inflammatory state associated with larger infarct volumes and carotid atherosclerosis, potentially justifying more aggressive anti-inflammatory interventions or targeted therapies. Conversely, the increased WBC counts in PC strokes may indicate a distinct immunologic mechanism, potentially involving variations in vascular architecture, blood-brain barrier permeability, or immune cell recruitment. These insights provide a rationale for exploring biomarker-guided, stroke subtype–specific anti-inflammatory strategies.

Further, the observed differences in vascular risk factor patterns—specifically, the lower prevalence of atrial fibrillation in PC strokes and the higher rate of prior stroke events—carry important implications for both primary and secondary prevention. These patterns suggest alternative underlying mechanisms, such as local vertebrobasilar pathology, and support the tailoring of prevention protocols to the specific risk profiles of each stroke subtype. This individualized approach could involve more intensive atrial fibrillation screening and management for patients at risk for AC strokes, and heightened surveillance or intervention for vertebrobasilar disease among those at risk for PC strokes.

Together, these findings support a fundamental shift toward precision medicine in stroke care. By integrating demographic variables, risk factor patterns, and biological markers into assessment and treatment strategies, stroke management can evolve beyond generalized protocols toward tailored interventions that reflect the specific pathophysiology of each patient and stroke subtype.

These evolving approaches to individualized care reflect a broader shift in clinical neuroscience. They exemplify the core principles of translational medicine, bridging mechanistic insights into inflammation and stroke subtype biology with practical, patient-centered applications. Further, the observed differences in vascular risk factor patterns—specifically, the lower prevalence of atrial fibrillation in PC strokes and the higher rate of prior stroke events—carry important implications for both primary and secondary prevention.

### 4.6 Study limitations

First, the study is characterized by imbalanced cohort sizes, with a smaller number of patients in the posterior circulation (PC) group (*n* = 65) compared to the anterior circulation (AC) group (*n* = 434). While this distribution, where PC strokes comprise approximately 13% of our cohort, is broadly consistent with established epidemiological data indicating that PC strokes account for 20%–25% of all ischemic strokes (8–10), we acknowledge that unequal group sizes can impact statistical power and the stability of multivariable models. However, it is a tenet of statistical interpretation that achieving a high level of statistical significance (*p* < 0.001) with a smaller sample size points toward a large and clinically meaningful effect size.

A subtle effect would likely have been missed, whereas the robust signal we detected for our primary inflammatory markers suggests a genuine and strong biological divergence rather than a chance finding vulnerable to low power. Furthermore, the paradoxical nature of our results—higher WBC counts in the PC group, which presented with lower clinical severity scores (NIHSS)—provides compelling evidence that these findings represent a genuine, location-specific inflammatory signature rather than a statistical artifact of sample imbalance. Nevertheless, we concur that the imbalance necessitates caution when interpreting the logistic regression models, as the coefficients for PC-specific predictors may be less stable. Our main conclusions, however, are anchored in the strong univariate findings.

Second, the retrospective, single-center nature of our study imposes important limitations but also offers distinct advantages for hypothesis generation. The retrospective design inherently limits our ability to establish causality and control for unmeasured confounding variables. Similarly, the single-center design constrains the direct external validity, or generalizability, of our findings. However, we contend that the principal contribution of this study lies in its high internal validity, which is often a strength of single-center research. By operating within a single institution with standardized diagnostic protocols and consistent laboratory techniques, we minimized extraneous variability, thereby enhancing our ability to isolate a clear and statistically robust biological signal. This controlled environment was critical for uncovering the novel, divergent inflammatory profiles—a finding that could have been diluted or masked in a more heterogeneous multicenter setting.

Therefore, this study should be interpreted as foundational, hypothesis-generating research. While the precise quantitative results may not be directly transferable, the fundamental hypothesis we propose—that AC and PC strokes elicit distinct biological responses (a systemic, CRP-dominant profile vs. a localized, leukocyte-dominant one)—is what holds significant potential for generalization. Furthermore, our study provides crucial, high-resolution data for a specific and underrepresented demographic in Northern Israel, including a substantial proportion of Arab patients. This focus aligns with previous work by our group that has specifically explored ethnic and gender variations in stroke patterns among this population (33), reinforcing the value of our regionally-focused data. The clear imperative now is for future large-scale, prospective, multicenter studies to test and validate this specific hypothesis across diverse ethnic, clinical, and healthcare settings, thereby moving from exploratory findings to confirmed, generalizable evidence.

### 4.7 Future research directions

Building on the limitations outlined above, the findings of this study point to several key directions for future research, with the aim of advancing personalized medicine approaches in stroke care. First, there is a clear need to develop and validate assessment tools that are specifically designed to capture the unique clinical features of posterior circulation (PC) strokes. Given the well documented limitations of the NIHSS in this context, future instruments should include evaluation of neurological functions that are often impaired in PC strokes—such as balance, coordination, visual fields, and cranial nerve function—but are underrepresented in current scales.

In addition, large-scale, multicenter prospective studies are essential to confirm and expand upon our results. Such studies should include diverse and balanced patient populations and employ standardized protocols for longitudinal data collection. Prospective designs would enable a more accurate characterization of temporal relationships between inflammatory markers, stroke severity, clinical course, and outcomes.

Further investigation into the genetic and molecular underpinnings of the observed differences between AC and PC strokes is also warranted. Exploring genome-wide associations, proteomic signatures, and inflammatory signaling pathways may help identify novel biomarkers and therapeutic targets, supporting a more precise and mechanistic understanding of stroke subtypes.

Moreover, future efforts should focus on the construction and validation of predictive models that integrate multiple patient-specific variables—including demographic characteristics, vascular risk factors, inflammatory markers (such as CRP and WBC), and genetic predispositions. These models could enhance individualized risk stratification and inform targeted prevention strategies tailored to stroke subtype. Pursuing these lines of investigation not only addresses current knowledge gaps but also supports the development of integrative clinical tools.

This approach exemplifies the goals of translational medicine—converting laboratory insights into applicable strategies for improving stroke care.

Finally, long-term follow-up studies are needed to assess the impact of these factors on functional recovery, recurrence, and quality of life. Such evidence would provide a more comprehensive view of stroke trajectories and help refine therapeutic approaches. Importantly, these efforts should culminate in prospective clinical trials evaluating the efficacy of personalized treatment protocols based on stroke subtype-specific characteristics. Taken together, these research directions represent essential steps toward closing existing knowledge gaps and establishing evidence-based, individualized care pathways for patients with ischemic stroke.

## 5 Conclusion

This study offers a comprehensive delineation of the fundamental clinical, biological, and demographic features distinguishing anterior and posterior circulation ischemic strokes. Our findings establish that AC and PC strokes constitute distinct pathophysiological entities, each associated with specific risk factor profiles, inflammatory responses, and clinical presentations. These distinctions underscore the limitations of current uniform approaches to stroke assessment and management, and support a transition toward personalized strategies tailored to stroke subtype.

The identification of elevated CRP levels in AC strokes and increased WBC counts in PC strokes highlights divergent inflammatory signatures, suggesting subtype-specific immunological processes. Additionally, differences in age and sex distribution—namely older age and male predominance in PC strokes—as well as the distinct pattern of vascular risk factors, including lower prevalence of atrial fibrillation and higher incidence of prior stroke in PC cases, further reinforce the need for refined risk stratification.

This distinction, grounded in measurable biological and clinical parameters, highlights a translational potential—linking pathophysiological mechanisms to more precise diagnostic and therapeutic strategies in stroke care.

Furthermore, our findings also reveal that patients with PC strokes present with significantly lower NIHSS scores, despite substantial neurological impairment. This discrepancy reflects a key limitation of standard assessment tools and emphasizes the need for more sensitive instruments capable of capturing posterior-specific symptoms, such as cranial nerve deficits, ataxia, and visual disturbances.

Taken together, these findings provide a strong foundation for the development of targeted diagnostic tools, prognostic models, and therapeutic interventions that align with the unique characteristics of each stroke subtype. By moving beyond generalized protocols and embracing a precision medicine paradigm, stroke care can become more responsive to individual patient profiles—enhancing outcomes across the continuum from acute treatment to secondary prevention and rehabilitation.

Beyond reinforcing existing knowledge, this study offers novel insights by directly contrasting inflammatory markers, clinical severity scores, and risk factor distributions across a large cohort stratified by stroke location. Specifically, the discovery of a divergent inflammatory signature—a CRP-dominant response in AC strokes versus a leukocyte-dominant response in PC strokes— represents a significant step toward redefining how stroke subtypes are assessed and managed from a biological standpoint. By integrating these multidimensional findings, the study lays the groundwork for datadriven, subtype-specific innovations in stroke diagnosis, treatment, and prevention.

## Data Availability

The datasets presented in this article are not readily available due to privacy considerations and ethical restrictions. However, de-identified data may be made available from the corresponding author upon reasonable request, subject to appropriate data sharing agreements and institutional approvals. Requests to access the datasets should be directed to SB, samihB@gmc.gov.il.
